# Development of Thermo-Responsive and Salt-Adaptive Ultrafiltration Membranes Functionalized with PNIPAM-co-PDMAC Copolymer

**DOI:** 10.3390/membranes15060164

**Published:** 2025-05-28

**Authors:** Lauran Mama, Johanne Pirkin-Benameur, Vincent Bouad, David Fournier, Patrice Woisel, Joël Lyskawa, Karim Aissou, Damien Quemener

**Affiliations:** 1 Institut Européen des Membranes, IEM-UMR 5635, Univ Montpellier, ENSCM, CNRS, 34090 Montpellier, France; lauran.mama@umontpellier.fr (L.M.); johanne.pirkin-benameur@umontpellier.fr (J.P.-B.); karim.aissou@umontpellier.fr (K.A.); 2 Univ. Lille, CNRS, INRAe, Centrale Lille, UMR 8207—UMET—Unité Matériaux et Transformations, 59000 Lille, France; vincent.bouad2@univ-lille.fr (V.B.); david.fournier@univ-lille.fr (D.F.); patrice.woisel@univ-lille.fr (P.W.); joel.lyskawa@univ-lille.fr (J.L.)

**Keywords:** membrane, thermo-responsive, PNIPAM, antifouling, stimuli-responsive, salinity

## Abstract

Access to clean water remains a critical global challenge, exacerbated by population growth, industrial activity, and climate change. In response, this study presents the development and characterization of thermo-responsive and salt-adaptive ultrafiltration membranes functionalized with a poly(N-isopropylacrylamide)–co-poly(dimethylacrylamide) (PNIPAM-co-PDMAC) copolymer. By combining the thermo-responsive properties of PNIPAM with the hydrophilic characteristics of PDMAC, these membranes exhibit dual-stimuli responsiveness to temperature and ionic strength, allowing for precise control of permeability and fouling resistance. The experimental results demonstrated that the copolymer’s hydration state and dynamic pore size modulation are sensitive to changes in salinity and temperature, with sodium chloride (NaCl) significantly influencing the transition behavior. Preliminary fouling tests confirmed the antifouling capabilities of these membranes, with salt-triggered hydration transitions effectively reducing irreversible fouling and extending membrane durability. The membranes’ reversible properties and adaptability to dynamic operating conditions highlight their potential to enhance the efficiency and sustainability of water treatment processes. Future investigations will focus on scaling up the fabrication process and assessing the long-term stability of these membranes under real-world conditions. This study underscores the promise of smart membrane systems for advancing global water sustainability.

## 1. Introduction

The lack of access to clean water is among the persistent challenges that humanity faces in the 21st century. This phenomenon has been accentuated by swift increases in global population, increased levels of industrial activities, as well as climate change. All of these have cumulatively contributed to the scarcity of fresh water resources, easily undermining the ability of billions of individuals to access safe, clean water [1]. The severity of the crisis is not only limited to the potential adverse effects on human health and sanitation but even on agricultural productivity, industrial growth, as well as conflicts between nations over shared water sources [2]. In such situations, it becomes necessary to develop new ways of treating water in order to address the problem of access to clean water on a global scale.

Membrane-based separation technologies have emerged as one of the most promising and energy-efficient methodologies to mitigate the global water crisis. In the past few decades, membranes have proved to be vital in filtration processes, desalination, and the purification of brackish and wastewaters due to the advantages they provide, such as components that take up less space and are easy to operate, scalable, and less energy demanding compared to water traditional methods [3]. In industrial and municipal applications, membrane techniques, such as reverse osmosis (RO), nanofiltration (NF), ultrafiltration (UF), and forward osmosis (FO), have gained significant popularity due to their capability to remove salt, pathogens, and other organic substances from water [4]. However, technological advancements have fallen behind despite significant progress in improving membrane performance in terms of higher permeability, selectivity, and chemical stability. Fouling, the process by which undesirable elements accumulate on the membrane surface, is one of several challenges that still exist. Indeed, this problem is one of the most serious, since it not only shortens the membrane lifespan but also raises operating expenses dramatically [5]. High selectivity and permeability can be reached without damaging the integrity of the membrane; however, this needs to be achieved without misestimating the engineering requirements [6]. Therefore, the need for membranes with properties that can be modified on demand and resist fouling is becoming more and more strong.

Since they can alter their structure and characteristics on demand, stimuli-responsive membranes, also referred to as “smart membranes”, are viewed as a great opportunity to revolutionize the water purification industry. These membranes exhibit the potential to change their permeability, pore size, and surface chemical properties in response to diverse factors such as temperature, pH, ionic strength, light, and electric fields [7]. This concept of external stimulation of membrane properties has interested researchers, as it brings in some level of adaptiveness and structural control, which was impossible with traditional membranes [8]. By adjusting operational conditions, one may be able to control fouling, achieve a better selectivity to solutes of interest, and possibly improve the durability of the membrane. For instance, heating or cooling a membrane incorporating a temperature-responsive polymer will cause the membrane to go from a hydrophilic highly permeable state to a hydrophobic one. This could enable the separation of complex mixtures, precise release, and effective cleaning of membranes. Membranes responsive to ionic strength or pH act similarly by altering the charge density, allowing for selective transport of ions and removal of certain contaminants depending, for instance, on the pH used.

Among stimuli-responsive polymers, poly(*N*-isopropylacrylamide) (PNIPAM) stands out due to its well-defined lower critical solution temperature (LCST), which is near physiological conditions (~32 °C). PNIPAM-based membranes are extremely adaptable materials for cutting-edge membrane technologies because of their exceptional thermosensitivity, which enables them to accurately adjust their permeability, wettability, and separation efficiency.

PNIPAM is integrated into membranes using a variety of methods, such as grafting, blending, and self-assembly [9,10,11,12]. Applications like oil–water emulsion separation [13] and the elimination of organic contaminants [14,15] demonstrate the practical usefulness of PNIPAM-based membranes. In addition, to improve the separation efficiency, the temperature-dependent shift between hydrophilic and hydrophobic states also confers antifouling properties [16]. For example, under thermal actuation [17], PVDF membranes combined with SiO_2_–PNIPAM particles have shown excellent oil separation performance and self-cleaning properties.

Furthermore, the pore size and permeability of thermo-responsive membranes can be dynamically changed, increasing their use in fields including protein adsorption, molecular sieving, and controlled drug delivery. PNIPAM-based membranes produce synergistic thermal reactions when paired with other materials, like graphene oxide, which improve material stability and separation performance [18].

One aspect that remains to be thoroughly investigated is the response of PNIPAM membranes to ionic strength. It has been demonstrated that the LCST of PNIPAM can shift in the presence of salts [19,20]. The dual-response system that can be created using PNIPAM membranes is interesting, as both temperature and ionic strength can influence the characteristics of the solution being filtered. While ionic strength-sensitive membranes do exist, they are primarily based on the electrostatic screening effect observed in charge-bearing membranes [21,22]. An interesting study has been published in this regard, demonstrating the use of salts to lower the LCST, with the aim of altering the pore size of the membrane [23]. The authors modified commercially available microporous polyvinylidene fluoride (PVDF) membranes through in situ thermal polymerization of vinyllactam monomers, enhancing their functionality for protein bioseparation. According to the results, the membranes can dynamically change the size of their pores in response to external stimuli like the concentration of salt. This property allows for more efficient separation processes, adapting to varying feed conditions.

In this work, commercial ultrafiltration (UF) membranes were coated with a statistical copolymer of PNIPAM and poly(dimethylacrylamide) (PNIPAM-co-PDMAC). The incorporation of dimethylacrylamide units, which are more hydrophilic than NIPAM, slightly raises the cloud-point temperature (T_cp_) of the thermosensitive polymer, ensuring it remains sufficiently above room temperature to allow for precise control during operation. The properties of these stimuli-responsive membranes are subsequently evaluated under reversible temperature variations and in the presence of salt (NaCl), highlighting their adaptability to dynamic environmental conditions. Particular emphasis is placed on characterizing permeability and surface properties and conducting preliminary fouling tests to assess the membranes’ functional performance. This work, therefore, targets the fabrication of temperature- and salt-responsive UF membranes that can actively mitigate fouling events; the consequent extension of service life, while anticipated, will be assessed in future long-term trials.

## 2. Materials and Methods

### 2.1. Materials and Chemicals

Unless otherwise noted, all reagents were acquired from Sigma-Aldrich (Saint Louis, MO, USA) and utilized without additional preparation. Hexane and methanol were used to recrystallize *N*-isopropylacrylamide (NIPAM) and 2,2′-azobis(isobutyronitrile) (AIBN), respectively. To remove the inhibitor, dimethylacrylamide (DMAC) was purified over silica. Before being used, dimethylformamide (DMF) was held in a nitrogen environment after being distilled over calcium hydride. Hexane, acetone, ethanol, diethyl ether, sodium sulfate, sodium chloride, and sodium bromide were all purchased from VWR (Fontenay-sous-Bois, France) and used as received. The process outlined in the literature was followed in the synthesis of the dopamine-based chain transfer agent (DOPA-CTA) [24]. Commercial polyethersulfone membranes (Millipore^®^, PBHK06210, Saint Quentin Fallavier, France) with an MWCO of 100 kDa and a diameter of 63.5 mm were immersed in MilliQ water for one hour, with the water being changed three times before use.

### 2.2. Synthesis of P(NIPAM-co-DMAC)-DOPA

RAFT (reversible addition fragmentation chain transfer) polymerization was used to prepare the copolymer. In a Schlenk tube, NIPAM (1 g, 8.83 mmol), DMAC (0.58 g, 5.89 mmol), AIBN (6 mg, 3.65 · 10^−5^ mol), and the chain transfer agent (DOPA-CTA, 71.2 mg, 0.184 mmol) were added in dry DMF (3 mL). The mixture was deoxygenated by argon bubbling for 30 min and then heated to 80 °C for 2 h. The polymer was purified by precipitation in hexane and diethyl ether. The ratio PNIPAM/DMAC was determined by ^1^H NMR (Bruker spectrometer operating at 300 MHz, Billerica, MA, USA) as 60/40 with DP = 80 and Mn_NMR_ = 8980 g.mol^−1^; M_n,SEC_ = 6200 g.mol^−1^ and Đ = 1.4 of P(NIPAM_48_-co-DMAC_32_)-DOPA were assessed using size exclusion chromatography (SEC, Agilent 1260 infinity equipped with a RI detector (Optilab) from Wyatt (Santa Barbara, CA, USA)), with DMF as the eluent, and poly(methyl methacrylate) standards were used for calibration. The T_cp_ = 55 °C of P(NIPAM_48_-co-DMAC_32_)-DOPA was determined by turbidimetry experiments with a Cary 3500 Multicell UV–Vis spectrometer from Agilent (Santa Clara, CA, USA).

### 2.3. Membrane Functionalization

Commercial polyethersulfone UF membranes were first coated with a thin intermediate layer of polydopamine (PDA). First, dopamine is added in MilliQ water to reach 0.1 mg/mL solution with 1:10 (*v*/*v*) of commercial Tris-Glycine buffer pH = 8.5: MilliQ water. The membrane is quickly immersed vertically into the solution and stirred at 70 rpm for 15 min. Once the reaction is finished, the membrane is washed directly with MilliQ water, so as not to let it dry, and then rinsed in an ultrasound bath for 2 min. The membrane is then stored in MilliQ water/ethanol 95:5 (*v*/*v*). Once the PDA layer has been deposited, a 0.5 M solution of PNIPAM_48_-co-PDMAC_32_ is prepared in MilliQ water with 1:10 (*v*/*v*) of commercial Tris-Glycine buffer at pH = 8.5 MilliQ water. The PDA-coated membrane is placed in the solution, and the reaction is performed at 35 °C for 30 min in a closed flask. Once the reaction is finished, the membrane is rinsed with MilliQ water and then stored in MilliQ water/ethanol 95:5 (*v*:*v*).

### 2.4. Membrane Characterization

**Contact angle analysis:** The membrane is dried in an oven at 30 °C for 6h before being taped to a support so that it is flat during the measurement. The measurement is made with the ILMS GBX and the Digidrop GBX software (Windrop++v1). The acquisition of the contact angle is performed manually, with a drop volume around 1.5 µL. A delay of 10 s is respected if the sample allows it before making the measurements. About 10 measurements are made before being averaged for each trial, and three trials are performed.

**Scanning electron microscope analysis**: A sample of the membrane is dry under vacuum to be analyzed and arranged on an analysis pad before being characterized with a Hitachi S4500 to analyze the surface morphologies. To analyze the cross-section of the membrane, samples are frozen in liquid nitrogen and then cut while the sample remains in the nitrogen. Surface porosity is obtained from binarized SEM images using Image J^®^ software (version 1.54p) with $\phi =\frac{{Area}_{pores}}{{Area}_{total\;surface}}\times 100$.

**X-ray Photoelectron Spectroscopy analysis:** Analysis of the dry membrane surface is performed by XPS to see the atomic concentration percentage of the carbon, oxygen, and nitrogen for different chemical conditions with a Thermo Electron’s ESCALAB 250 analyzer (Thermo Fisher Scientific, Waltham, MA USA). The source of excitation is ray Al K (1486.6 eV), a monochromatic source. The diameter of the surface under analysis is 500 µm. The photoelectron spectrums are calibrated in relation to the energy of the C-C component of carbon 1s, which is 284.8 eV. A low-energy electron beam (−2 eV) is used to balance the charge.

**Transmittance measurement:** The measurements are made using a dynamic light scattering analysis on a Malvern Zetasizer Nano ZS instrument (Worcestershire, United Kingdom) with a 4 mV He-Ne laser operating at = 633 nm. The concentration of the PNIPAM_48_-co-PDMAC_32_ aqueous solution is fixed at 1 mg/mL. Quartz cuvettes are used to analyze the samples.

### 2.5. Water Permeation

Filtration experiments for this study used dead-end filtration mode with a homemade filtration cell (diameter = 25 mm). A 1L Amicon tank filled with MilliQ water was connected to the cell. For all the flux trials, the pressure was kept constant at 1 bar. To fit into the slot of the stirred cell, the membranes were sliced into circular pieces of 2.5 cm in diameter. Permeated water was measured using a balance connected to the S232 Data Logger software (2.6.0), with a 0.599 s time step acquisition. Before conducting any filtration measurements in this study, the membrane was placed in the cell, compacted progressively from 1 bar to 4 bar (increments of 1 bar every 15 min), and held for 1 h at 4 bar; then, the pressure was lowered to 1 bar for all subsequent measurements. Compaction was conducted at 25 ± 1 °C to prevent premature collapse of the PNIPAM-co-PDMAC layer and to isolate mechanical consolidation of the PES support from thermo-responsive effects.

### 2.6. Antifouling Evaluation

This study investigated the antifouling properties of the prepared ultrafiltration (UF) membranes by employing a model foulant, bovine serum albumin (BSA at 0.1 g/L), simulating protein fouling. Three dynamic fouling cycles were conducted per membrane. Initially, the membrane underwent compaction with deionized (DI) water at 1 bar pressure for 30 min, during which the initial water flux (J_0_) was recorded. Subsequently, the system was exposed to a freshly prepared BSA solution at the same pressure for 15 min, and the resulting BSA flux (J_BSA_) was measured. Post-filtration, the membrane underwent either deionized water or saline water washes without applied pressure for 30 min each. Finally, the system was switched to pure deionized water at 1 bar pressure, and the next cycles were repeated following the same procedure.

## 3. Results and Discussion

### 3.1. Preparation of Responsive PES Commercial Membranes

**Figure 1:**
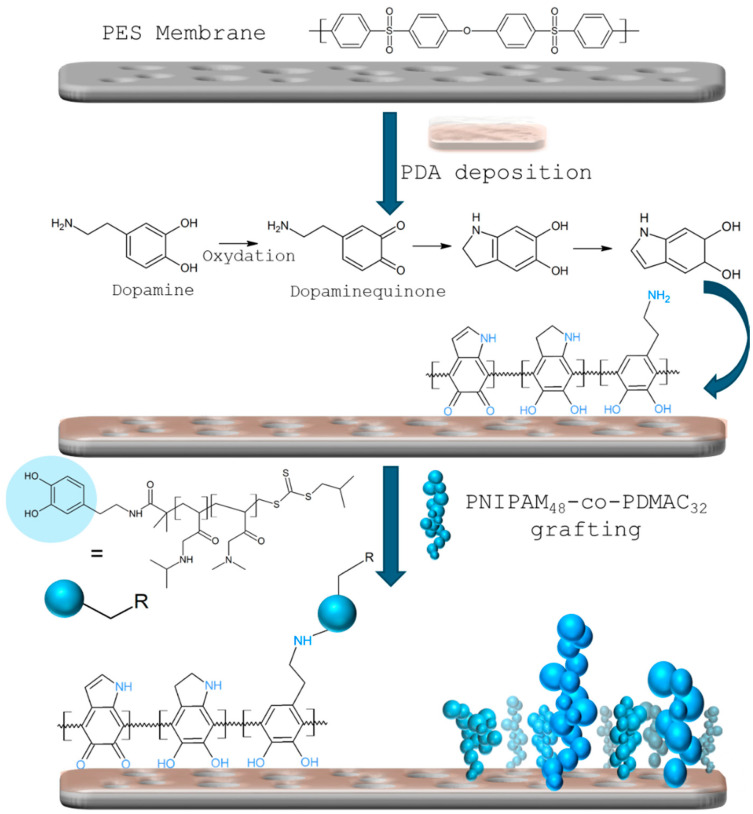
Schematic illustration of poly(N-isopropylacrylamide)–co-poly(dimethylacrylamide) (PNIPAM-co-PDMAC) membrane grafting.

In order to prepare responsive membranes, a random copolymer based on N-isopropylacrylamide (NIPAM) and N, N-dimethylacrylamide (DMAC) units was firstly synthesized by reversible addition-fragmentation chain transfer (RAFT) copolymerization of NIPAM and DMAC using a dopamine functionalized chain transfer agent (DOPA-CTA), the latter being responsible for the further grafting of the thermo-responsive copolymer onto the membrane (Figure 1). A molecular weight of 6200 g.mol^−1^ with a low dispersity of 1.4 were obtained for the P(NIPAM_48_-co-DMAC_32_)-DOPA copolymer. It is noteworthy that the degree of polymerization, around 80, was selected based on previous studies that highlighted sufficient conformational change to impact pore size and, hence, the transmembrane flux of the membranes [25].

As depicted in Figure 1, our strategy to graft the thermosensitive PNIPAM_48_-co-PDMAC_32_ copolymer onto commercial polyethersulfone (PES) membranes first involved the deposition of a thin layer of polydopamine (PDA) onto the commercial PES membrane surface. Dopamine self-polymerizes to create thin, surface-adherent polydopamine films on various inorganic and organic materials, which can subsequently undergo secondary reactions to form diverse adlayers [26]. Grafting of PNIPAM_48_-co-PDMAC_32_ was achieved by an aza-Michael addition between the quinone form of its terminal catechol and the residual primary amines of the PDA coating (Figure 1) [27]. The “grafting onto” technique is preferred over “grafting from” due to the latter’s susceptibility to side reactions during polymerization, leading to a higher dispersity of chains and thus affecting thermosensitivity. Covalent grafting is also favored over physisorption to extend the coating’s durability and effectively orient the copolymer chains on the surface, maximizing the amplitude of conformational changes.

**Figure 2:**
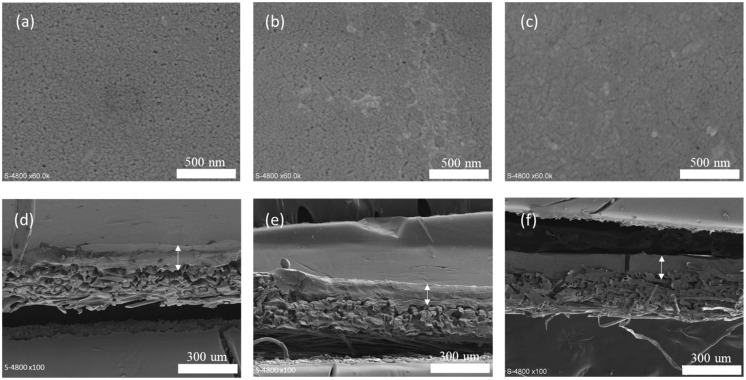
SEM images of the surface of (**a**) commercial membrane, (**b**) PDA-coated membrane, and (**c**) copolymer-grafted membrane as well as cross-sections of (**d**) commercial membrane, (**e**) PDA-coated membrane, and (**f**) copolymer-grafted membrane.

Scanning electron microscopy (SEM) was used to analyze the successive stages of membrane modification (Figure 2). While minimal alterations were observed on the surface morphology following functionalization, surface porosity calculations indicate a reduction from 26 ± 4% for the commercial PES membrane to 18 ± 3% for the PDA-coated membrane and, further, to 12 ± 4% for the grafted membrane. The thickness of the selective layer, approximately 90 ± 10 nm, does not vary significantly. In the SEM images, the arrows indicate the membrane, while the remaining non-woven substructure serves only as a mechanical support.

**Figure 3:**
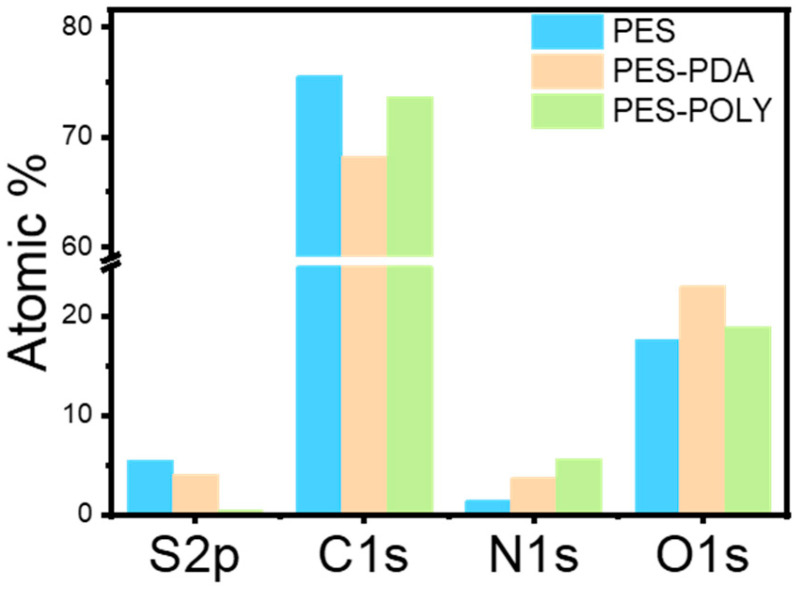
XPS atomic percentages for PES commercial membrane, PES-PDA, and PES-POLY.

Figure 3 displays X-ray photoelectron spectroscopy (XPS) atomic percentages for the commercial PES membrane (PES), PDA-coated PES membrane (PES-PDA), and PNIPAM_48_-co-PDMAC_32_ grafted PES-PDA membrane (PES-POLY). XPS analysis confirms the presence of a coating on the PES membrane, as indicated by the decrease in sulfur percentage, which is mainly present in the commercial membrane, after each step. Another important observation is the increase in nitrogen percentage when the membrane is coated with PDA, which is further enriched after grafting the nitrogen-rich PNIPAM_48_-co-PDMAC_32_ copolymer.

### 3.2. Hydrophilicity and Water Permeability of Responsive PES Commercial Membranes

**Figure 4:**
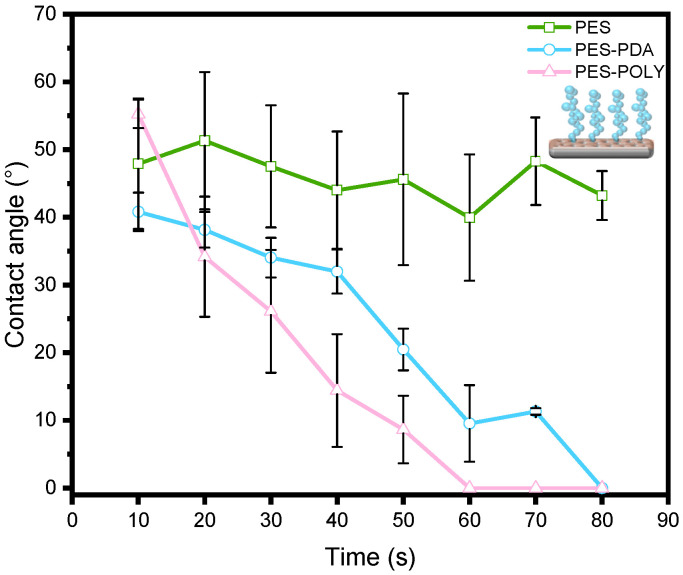
Water contact angle measurements of PES commercial membrane (PES), PDA coated membrane (PES-PDA), and PNIPAM_48_-co-PDMAC_32_ grafted membrane (PES-POLY).

The influence of PDA and PNIPAM_48_-co-PDMAC_32_ coatings on the hydrophilicity of the PES membrane was assessed through water contact angle (WCA) measurements (Figure 4). WCA was measured every 10 s over 80 s to observe variations resulting from the porous structure of the membranes. Two concurrent effects are noted here: the surface tension of the membrane’s extreme surface, defining the hydrophilicity of the materials, and the membrane’s pore size, which, beyond a threshold value, can allow for water penetration into the material, leading to an apparent decrease in the contact angle. The commercial PES membrane exhibits a WCA of approximately 46 ± 4°, which remains stable over the observed period. This angle indicates a rather hydrophilic surface, and the lack of change over time suggests the absence of spontaneous water absorption by the porous material. After coating with a PDA layer, the initial contact angle remains close to that of the precursor membrane; however, a rapid decrease is observed, reaching 0° after 80 s. This suggests that, although the pore size likely decreased after PDA functionalization (as demonstrated later), the threshold pore size for water penetration into the material was significantly lowered due to the hydrophilic nature imparted by the PDA [28]. After PNIPAM_48_-co-PDMAC_32_ grafting, the same dynamics are preserved, indicating the hydrophilic nature of the final membranes.

If the measurement of the WCA allows for an evaluation of macroscopic surface properties, the measurement of water permeance also enables the monitoring of membrane pore evolution during functionalization. The initial permeance of the PES commercial membrane (215 LMH/bar) decreases to 121 LMH/bar after PDA deposition, despite an increase in surface hydrophilicity (see WCA), indicating a reduction in surface porosity, in agreement with previous SEM analysis (see Figure 2). Permeance decreases slightly to 104 LMH/bar after functionalization with the copolymer, suggesting minimal pore size evolution during this final functionalization step, consistent with previous findings [25]. This suggests that the PNIPAM_48_-co-PDMAC_32_ membrane has strong wettability, which is thought to improve its antifouling and fouling release capabilities.

### 3.3. Influence of Thermosensitive Properties on Membrane Flux

**Figure 5:**
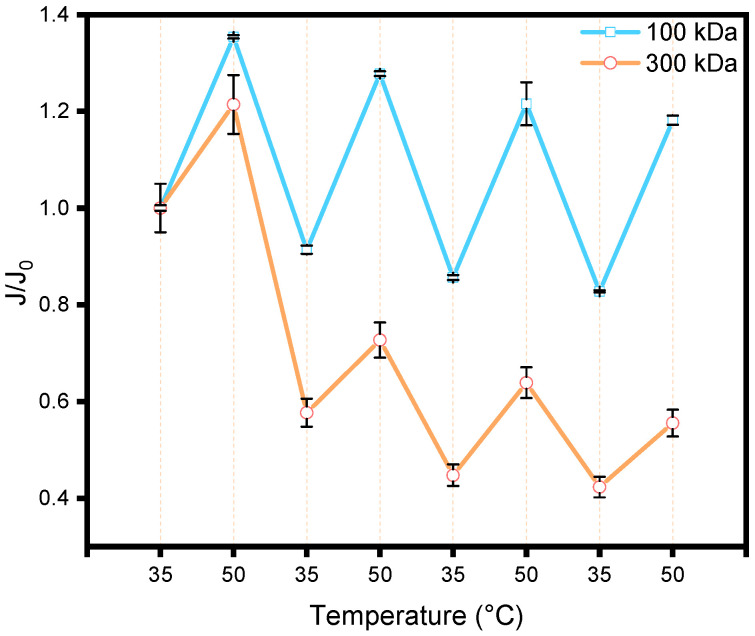
Measurement of the flux for 100 kDa and 300 kDa membrane grafted with PNIPAM_48_-co-PDMAC_32_ (PES-POLY) for 30 min at 35 °C and 50 °C.

Figure 5 shows the transmembrane flux of membranes grafted with PNIPAM_48_-co-PDMAC_32_ (PES-POLY,) at 35 °C and 50 °C, considering that the polymer has a transition temperature (T_cp_) around 40 °C after being grafted onto the membrane (shown later in part 3.4). The water flux was measured by placing the membrane in a dead-end filtration cell at a pressure gradient (ΔP) of 1 bar. Two commercial PES membranes with different molecular weight cut-offs (100 kDa and 300 kDa) were used. The measured water fluxes (150 LMH at 35 °C for 100 kDa and 448 LMH at 35 °C for 300 kDa) were different; hence, normalization by the initial flux at 35 °C was applied to facilitate data comparison. Three temperature cycles of 30 min, alternating between 35 °C (below T_cp_) and 50 °C (above T_cp_), were performed. The flux was also normalized by the water viscosity at the given temperature to account for the mechanical increase in flux due to decreased viscosity with rising temperature, independent of any membrane changes. Cyclic flux increases and decreases were observed in response to temperature changes, directly resulting from the thermosensitivity of the copolymer chains.

At 35 °C, below the T_cp_, the chains are hydrated and “extended” within the pores, whereas at 50 °C, the transition to a collapsed globular conformation frees space within the pores, thus increasing water flux. Notably, this transition also reduces the hydrophilicity of the pore surfaces due to the dehydrated state of the copolymer. However, the pore size seems large enough that this effect does not significantly counterbalance the observed increase in pore size, resulting in a flux increase. The cycles are reversible, with a slight damping for the 100 kDa grafted membrane (~10% in flux value after the 3rd cycle) and a more pronounced damping for the 300 kDa grafted membrane (~48% in flux value after the 3rd cycle). Furthermore, the flux cycle amplitude is greater for the 100 kDa membrane (40% flux variation) compared to the 300 kDa membrane (20% flux variation), possibly explained by the use of the same copolymer in both cases. The impact of chain volume change on pore size is more pronounced for smaller pores with constant chain length. For the 300 kDa membrane, the pronounced flux loss observed in the first cycle could represent a pre-conditioning step: the initial heat-collapse/cool-rehydration sequence may reset the brush conformation within the wider pores, after which cycles 2 and 3 appear to mirror the 100 kDa behavior, albeit with a lower thermo-responsive amplitude. Due to lower stability and more restricted flux cycle amplitude, the remainder of this study focuses on the 100 kDa membrane.

### 3.4. PNIPAM-co-PDMAC State in Solution and on the Membrane Surface

**Figure 6:**
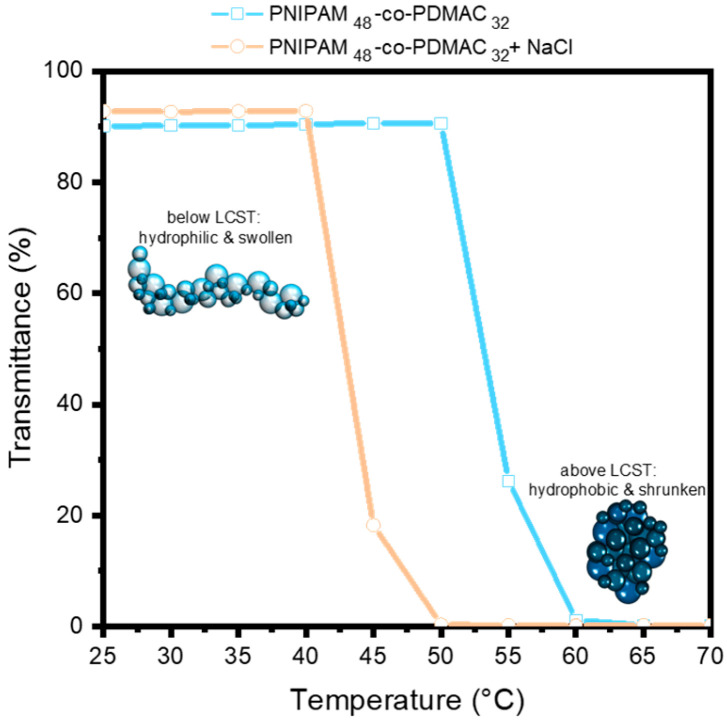
Measurement of the transmittance of aqueous PNIPAM_48_-co-PDMAC_32_ solutions (1 mg/mL) against the temperature in the presence and in the absence of NaCl ([NaCl] = 0.2 M).

It is well established that the inclusion of salts can modify the stability of the hydrated state of thermosensitive polymers, which consequently results in a decrease in their T_cp_ [29]. This phenomenon is corroborated by our investigation (Figure 6), wherein the turbidity of an aqueous copolymer solution (1 mg/mL) was evaluated as a function of temperature, both in the absence and presence of salt ([NaCl] = 0.2 M). The dehydration process of the copolymer transpires at a diminished T_cp_ (≈45 °C instead of 55 °C).

**Figure 7:**
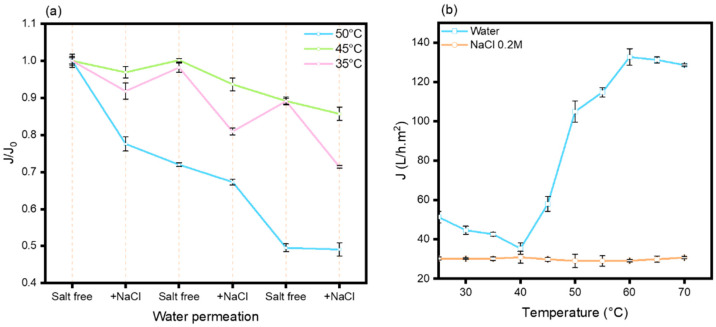
Membrane salt responsiveness. (**a**) Normalized flux of PNIPAM_48_-co-PDMAC_32_ grafted PES membrane during 30 min cycles at 35 °C, 45 °C, and 50 °C, with and without NaCl ([NaCl] = 0.2 M). (**b**) Normalized flux of PNIPAM_48_-co-PDMAC_32_ grafted PES membrane as a function of temperature with and without NaCl ([NaCl] = 0.2 M).

At a temperature situated between the two T_cp_ (with and without the presence of salt), a conformational alteration in the copolymer chains can be discerned without modification of the temperature, influenced solely by the presence of salt. The transition temperature recorded for unbound chains in solution may exhibit substantial discrepancies when compared to that determined for the identical chains grafted to a surface. The globular and collapsed conformations cannot be congruent due to the limitations imposed by the surface, where the chains are anchored at one end. The density of grafting and the accessible free space for each chain to evolve within a two-dimensional space diverge markedly from the dynamics observed in solution. Following the grafting of the copolymers onto the PES membrane that was first coated with PDA, cycles of water permeation assessments were conducted, both in the presence and absence of salt ([NaCl] = 0.2 M) at three distinct temperatures (35 °C, 45 °C, and 50 °C) (Figure 7a).

When evaluating normalized flux values, cyclical patterns are exclusively discernible at a temperature of 35 °C. Nevertheless, in contrast to prior hypotheses, the alteration from deionized water to saline solutions resulted in a decline in flux rather than an anticipated increase as previously discussed. A flux decrease throughout the cycles is particularly evident at 50 °C, which may be ascribed to the phenomenon of membrane compaction. Notwithstanding this observation, the amplitude of flux cycles at 35 °C remains constant or may even exhibit a slight increase, as shown by the calculation of amplitude variation, where an increase from 7% to 14% is observed. The consistency of the cycle amplitude suggests the potential for reversible conformational shifts within the copolymer chain structures.

To elucidate the phenomenon of flux inversion in the presence of sodium chloride, we carried out an investigation into the water permeation characteristics of the membrane from 25 to 70 °C, both in the presence and absence of NaCl ([NaCl] = 0.2 M) (Figure 7b). The water flux profile recorded in the absence of salt reveals a notable transition at approximately 47 °C, thereby underscoring the thermosensitive properties inherent to the membrane. Below this temperature threshold, the flux decreases with increasing temperature, which may be indirectly attributable to the initiation of copolymer dehydration. The thermally induced conformational alteration results in a substantial increase in flux (fourfold) between 40 °C and 60 °C, followed by a phase of stabilization. Conversely, in the presence of salt, the thermosensitivity of the membrane disappears within the measured temperature range. In contrast to the behavior observed in aqueous solution (where a decrease in the T_cp_ is noted with the addition of salt), the grafted copolymer chains fail to manifest any significant conformational alterations. Moreover, the initial flux is also lower.

As articulated by Uhlmann et al., in the sub-T_cp_ regime, a volumetric expansion of the polymer occurs subsequent to the introduction of salt, which is a result of intensified steric interactions among proximal segments [30]. This volumetric expansion, when coupled with the restricted nature of the membrane pores, seemingly inhibits any conformational modifications, even at temperatures 30 °C above the T_cp_. This phenomenon further elucidates the observed flux cycle inversion, as the flux of saline water is markedly inferior to that of pure water. Hence, the cycles observed are not attributable to a pronounced conformational transition from a hydrated globular state to a collapsed hydrophobic form but rather to a more nuanced conformational adjustment occurring within the hydrated state.

**Figure 8:**
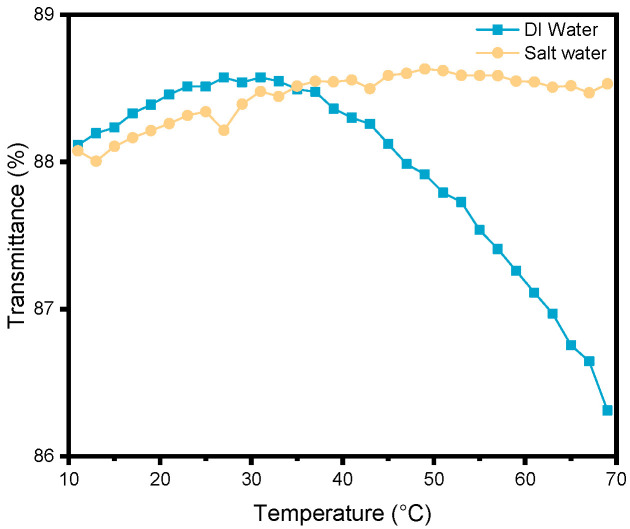
Transmittance of PNIPAM_48_-co-PDMAC_32_ grafted glass in deionized and saline water as a function of temperature.

An additional experiment was conducted to isolate the conformational change from the complexity introduced by the membrane’s porous structure (Figure 8). The copolymer was grafted onto a dense glass substrate, activated using O_2_ plasma treatment before undergoing functionalization with the same protocols used for PDA deposition and polymer grafting. The PNIPAM_48_-co-PDMAC_32_ grafted glass is then immersed in water (with or without salt). Transmittance through the glass was measured over a temperature range of 10 °C to 70 °C. In the presence of salt, thermosensitivity was confirmed to be absent, while a decrease in transmittance was observed starting at 35 °C, corresponding to the expected dehydration of the copolymer in pure water.

**Figure 9:**
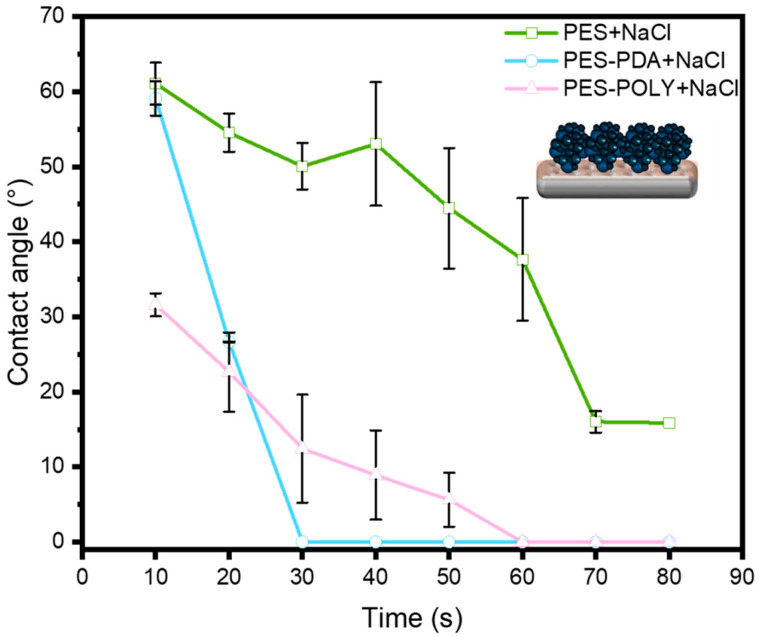
Saline water contact angle measurements of PES commercial membrane (PES), PDA coated membrane (PES-PDA), and PNIPAM_48_-co-PDMAC_32_ grafted membrane (PES-POLY).

Measuring the saline water contact angle on the membrane surfaces generally revealed greater wettability in the presence of salt (Figure 9). However, the values were comparable to those measured with pure water (Figure 4), confirming the hydrophilic nature of the surfaces and the hydrated state of the copolymers in the presence of salt. Ultimately, this absence of a conformational transition could be advantageous for fouling prevention, as it avoids the hydrophobic nature of the collapsed dehydrated PNIPAM-based copolymer while maintaining the membrane’s flux-stimulating response to temperature and salt presence.

To investigate whether the nature of the salt, as described by the Hofmeister series, influences the behavior of the grafted copolymer membrane, filtration cycles were conducted in the presence of a cation larger than Na⁺ (KCl) and a cation smaller than Na⁺ (LiCl). According to the Hofmeister series, kosmotropic cations like K⁺ tend to destabilize macromolecules, whereas chaotropic cations like Li⁺ tend to stabilize them [19]. Supplementary Figure S3 shows that ionic filtration cycles with LiCl ([LiCl] = 0.2 M) do not induce any flux variations. However, ionic filtration cycles with KCl ([KCl] = 0.2 M) produce larger flux variations compared to the NaCl experiments. Further work is needed to fully understand the effect of the Hofmeister series on filtration cycles. However, despite their smaller amplitude, NaCl–water cycles are preferable to KCl due to the widespread availability, lower cost, and established industrial use of NaCl.

### 3.5. Preliminary Results on Protein Removal Using Salt-Based Cleaning Cycles

Since a change in water flux was observed in response to the presence of NaCl, we aimed to test the cleaning capacity of this membrane in the presence of saline water ([NaCl] = 0.2 M) compared to deionized water. A fortuitous advantage provided by the suppression of the T_cp_ of the copolymer in the presence of salt in our system is that the copolymer chain remains hydrated and maintains the surface in a good hydrophilic state, favorable for limiting fouling, unlike the expected transition for a PNIPAM, which becomes hydrophobic above its T_cp_.

In this way, three filtration cycles of a model fouling agent, bovine serum albumin (BSA at 0.1 g/L), were performed. Each cycle included the same steps: conditioning the membrane for 30 min, filtering BSA for 15 min, and washing either with deionized water or saline water for 30 min. Conditioning, water permeation, and BSA filtration were carried out at a pressure drop of 1 bar, while membrane washing was performed at atmospheric pressure under agitation within the filtration cell.

**Figure 10:**
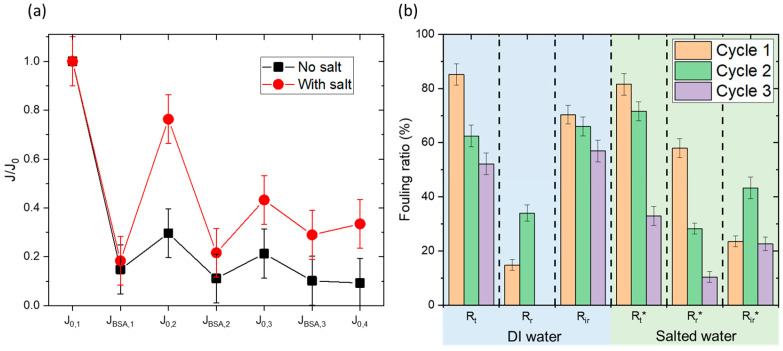
BSA removal using salt-based cleaning cycles. (**a**) Normalized flux cycles alternating water permeation and BSA filtration. Each cycle is followed by a membrane cleaning (DI water or saline water ([NaCl] = 0.2 M). (**b**) Corresponding fouling ratios at different cycles.

Figure 10a shows the flux cycles, normalized by the initial water flux (J_0,1_) to allow for comparisons. A clear trend emerges: after a significant drop in flux following BSA filtration in both cases, a greater recovery of the initial flux is observed during washing with NaCl.

For more detail, Figure 10b presents the fouling ratios in both cases, calculated using the following equations:

(1) $$R_{t}=\frac{J_{0,n}-J_{BSA,n}}{J_{0,n}}$$

(2) $$R_{r}=\frac{J_{0,n+1}-J_{BSA,n}}{J_{0,n}}$$

(3) $$R_{ir}=\frac{J_{0,n}-J_{0,n+1}}{J_{0,n}}$$

Here, J_0,n_ is the permeation flux of deionized water corresponding to cycle n, and J_BSA,n_ is the flux after BSA filtration in cycle n. R_t_ is the total fouling ratio, R_r_ is the reversible fouling fraction, and R_ir_ is the irreversible fouling fraction.

Initially, R_t_ values are comparable across experiments, as expected, given they are calculated during the same part of the cycle (conditioning and then BSA filtration). R_t_ is around 80%, indicating a significant 80% decrease in flux due to BSA filtration, signifying evident fouling. R_t_ decreases slightly with each cycle, which seems logical as residual fouling leaves less clean surface area available for further fouling. The key difference lies in the recovery of the original flux after membrane cleaning R_r_. While deionized water washing recovers only 15% of the initial flux in the first cycle, saline water washing recovers nearly 60%. Consistently, the irreversible fouling fraction (R_ir_) is 70% after deionized water washing and 24% after saline water washing. This trend is similar across subsequent cycles, with irreversible fouling fractions remaining significantly higher for deionized water compared to saline water.

The antifouling potential derived from the conformational change in a polymer chain at the membrane surface is well known, for instance, via temperature change as previously discussed. However, to our knowledge, cleaning efficiency enhanced solely by the presence of salt is unique for this type of membrane.

## 4. Conclusions

In conclusion, this study demonstrated the successful development and characterization of thermo-responsive and salt-adaptive ultrafiltration membranes functionalized with PNIPAM-*co*-PDMAC copolymers. By leveraging the unique thermo-responsive properties of PNIPAM and the hydrophilic characteristics of PDMAC, the membranes exhibit a precise and reversible response to both temperature variations and ionic strength. The inclusion of hydrophilic PDMAC units slightly raised the T_cp_ of the copolymer, enabling its operation at temperatures safely above ambient conditions, which ensures enhanced control over permeability and fouling resistance during practical applications. The experimental results showed that these membranes achieve dynamic pore size modulation in response to external stimuli. The ionic strength tests revealed that the presence of salts, such as NaCl, could lower the T_cp_. However, when grafted onto the UF membrane, T_cp_ is no longer measurable, but a change in the hydration state of the copolymer is observed. This dual-stimulus responsiveness provides a significant advantage in operational flexibility. Preliminary fouling tests underscored the antifouling potential of these membranes, as the salt-dependent copolymer transition allowed for reduced irreversible fouling. This property not only improves the long-term durability of the membranes but also decreases operational costs associated with cleaning and maintenance. In practical terms, the salt-induced rehydration of the grafted layer enables a simple rinse with a low-concentration NaCl solution to restore permeability and remove foulants, providing a cost-effective, chemical-free self-cleaning step between filtration cycles. Overall, this work highlights the potential of PNIPAM-based membranes to transform membrane technologies by introducing a level of adaptability and efficiency unattainable with traditional membrane systems. Despite these promising results, the present study was limited to 5 cm^2^ laboratory coupons evaluated in short-duration dead-end tests with a single model protein. Future studies should focus on scaling up the fabrication process and investigating, in detail, long-term cycling with real effluents. By addressing these areas, this research could pave the way for the broader adoption of smart membrane systems, contributing significantly to global water sustainability initiatives.

## Data Availability

The original contributions presented in this study are included in the article/Supplementary Materials. Further inquiries can be directed to the corresponding author.

## Supplementary Materials

The following supporting information can be downloaded at: https://www.mdpi.com/article/10.3390/membranes15060164/s1, Figure S1: High-resolution XPS spectra of C, O, N for the PES membrane, PDA membrane and the copolymer grafted membrane; Figure S2: Wide scan XPS spectra of the PES membrane, PDA membrane and the copolymer grafted membrane; Figure S3: Measurement of the flux of 100 KDa membrane grafted with PNIPAM-co-PDMAC for 30 min at 35°C, with and without different salts ([salt] = 0.2 M).
