# Supplementary material for: Development of Thermo-Responsive and Salt-Adaptive Ultrafiltration Membranes Functionalized with PNIPAM-co-PDMAC Copolymer
Source: Membranes (Basel). 2025 May 28;15(6):164. doi: 10.3390/membranes15060164 (PMC12195130; doi:10.3390/membranes15060164)
Supplement: Supplementary file 1 [file membranes-15-00164-s001.zip › membranes-3646703-supplementary.pdf]

# Supplementary Material

# Development of thermo-responsive and salt-adaptive ultrafiltration membranes functionalized with PNIPAM-co-PDMAC copolymer

**Lauran Mama[1], Johanne Pirkin-Benameur[1], Vincent Bouad[2], David Fournier[2], Patrice Woisel[2], Joël Lyskawa[2], Karim Aissou[1], Damien Quemener[1*]**

[1] *Institut Européen des Membranes, IEM-UMR 5635, Univ Montpellier, ENSCM, CNRS, 34090 Montpellier, France*

[2] *Univ. Lille, CNRS, INRAe, Centrale Lille, UMR 8207 - UMET - Unité Matériaux et Transformations, Lille, France.*

* **Corresponding author**: Prof. Damien Quemener, E-mail: damien.quemener@umontpellier.fr

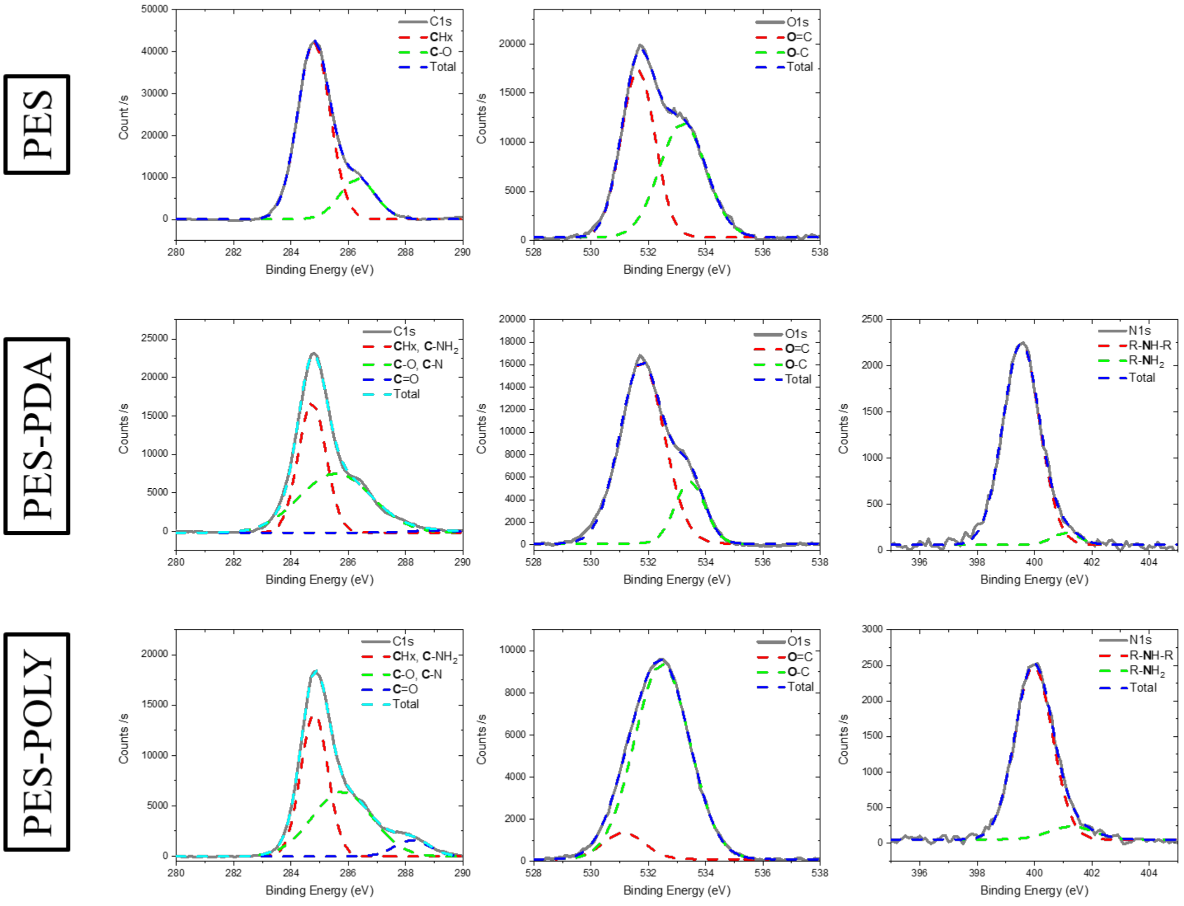


Figure S1: High-resolution XPS spectra of C, O, N for the PES membrane, PDA membrane and the copolymer grafted membrane.

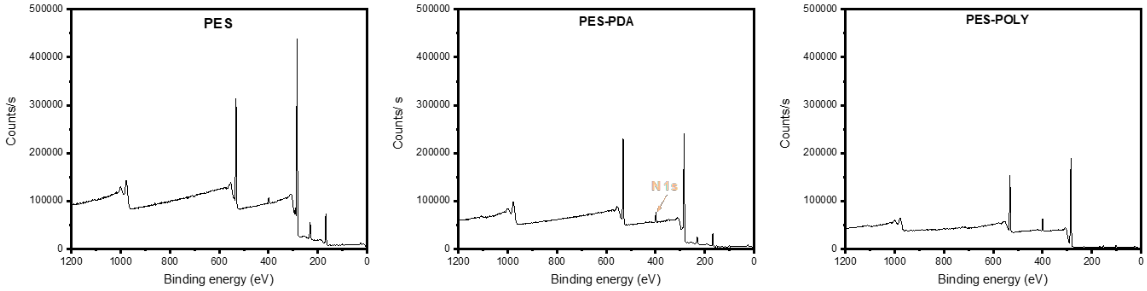


Figure S2: Wide scan XPS spectra of the PES membrane, PDA membrane and the copolymer grafted membrane.

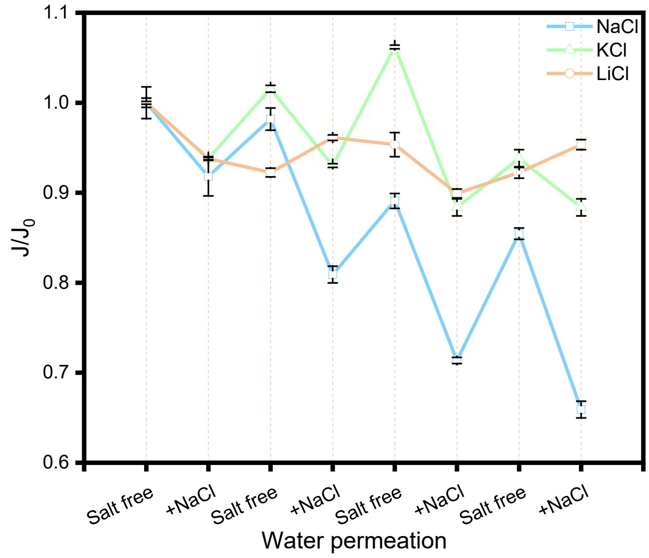


Figure S3: Measurement of the flux of 100 KDa membrane grafted with PNIPAM-co-PDMAC for 30 minutes at 35°C, with and without different salts ([salt]=0.2M).
